# Absorbable magnesium-based stent: physiological factors to consider for *in vitro* degradation assessments

**DOI:** 10.1093/rb/rbu015

**Published:** 2015-01-06

**Authors:** Juan Wang, Christopher E. Smith, Jagannathan Sankar, Yeoheung Yun, Nan Huang

**Affiliations:** ^1^Key Laboratory of Advanced Technologies of Materials, Ministry of Education, School of Materials Science and Engineering, Southwest Jiaotong University, Chengdu 610031, China and ^2^National Science Foundation Engineering Research Center for Revolutionizing Metallic Biomaterials, North Carolina A & T State University, Greensboro, NC 27411, USA

**Keywords:** magnesium, absorbable stent, *in vitro*, *in vivo*, biodegradation

## Abstract

Absorbable metals have been widely tested in various *in vitro* settings using cells to evaluate their possible suitability as an implant material. However, there exists a gap between *in vivo* and *in vitro* test results for absorbable materials. A lot of traditional *in vitro* assessments for permanent materials are no longer applicable to absorbable metallic implants. A key step is to identify and test the relevant microenvironment and parameters in test systems, which should be adapted according to the specific application. New test methods are necessary to reduce the difference between *in vivo* and *in vitro* test results and provide more accurate information to better understand absorbable metallic implants. In this investigative review, we strive to summarize the latest test methods for characterizing absorbable magnesium-based stent for bioabsorption/biodegradation behavior in the mimicking vascular environments. Also, this article comprehensively discusses the direction of test standardization for absorbable stents to paint a more accurate picture of the *in vivo* condition around implants to determine the most important parameters and their dynamic interactions.

## Introduction

With the development of regenerative biomaterials, absorbable metallic stents are being investigated as an alternative for permanent stents, which hold arteries open after percutaneous coronary intervention to maintain arterial blood flow [1–3]. Absorbable stents are expected to corrode gradually *in vivo*, with an appropriate host response elicited by released corrosion products, then dissolve completely upon fulfilling the mission to assist with tissue healing [4]. Potential benefits of absorbable stents include cessation or elimination of antiplatelet therapies that complicate the necessity for the treatment of other medical conditions the patients require—adding risk to the overall health [5]. Other possible benefits include the restoration of vessel properties such as vasomotion which is the ability of the vessel to expand and contract as the body attempts to modulate blood flow between times of rest and exercise. Additionally, absorbable stents would likely create the ability to more efficiently treat a previously stented site, or to enable the option of a bypass graft without the hindrance of an existing implant. Absorbable stents may also be used successfully as a way to deliver anti-restenosis drugs [6]. Absorbable stents made of Mg and its alloys have been studied for several years in clinical trials with reportedly encouraging results [6–14].

The accurate determination of bioabsorption for absorbable metals is a technologically important issue in science and biomedical applications [15]. However, there is a paucity of reports that focus on the correlation between *in vitro* and *in vivo* testing. It has been observed that corrosion rate *in vivo* is about four orders of magnitude slower than that *in vitro* with ASTM standard [16]. *In vitro* testing methods that accurately mimic the *in vivo* environment are required to improve the standardization process by which a material is deemed appropriate for more costly *in vivo* testing which further involves the welfare of animals.

Thorough knowledge of the comportment of Mg and its alloys in physiological environments is limited as a result of the adolescence in terms of information and research available in this area. Every medical device undergoes a common surface–environment interaction in which the device material is exposed to the biological environment that contains organic and inorganic constituents, followed by complex blood, cell and tissue response. Also, the fluid dynamics, gas diffusion, pH and temperature have led to differing impacts on the behavior of Mg at the early- and long-term implantation process. Evaluating and understanding the biological response and subsequent Mg behavior is vital in the development of absorbable metallic stents with controlled degradation rates and optimum biocompatibility. Of fundamental concern is the determination of relevant *in vitro* environmental setup parameters that approximate *in vivo* physiological parameters [17].

For the process of commercialization of absorbable Mg-based stent, it is mandatory to perform biodegradation and biological safety evaluations. Determining the most relevant *in vitro* bench tests as well as identifying the most important parameters to assess is a significant obstacle in spite of the international technical standards available (e.g. ASTM-G31-72 and ISO 10993 series [18]) [19, 20]. A new standard ISO/TR 37137:2014 was published about ‘Cardiovascular biological evaluation of medical devices - Guidance for absorbable implants’, which is to provide interim part-by-part guidance on potential adjustments to various test methods within the 10993 series to account for the intentional release of soluble components or degradation products from absorbable medical devices. For the success of commercialization of absorbable Mg-based stents, appropriate biodegradation testing condition still need to be improved to help screen the biocompatibility of developed Mg-based stents *in vitro*, which saves cost and testing periods comparing with those *in vivo*.

In this up-to-date review, we attempt to analyze the influence of physiological parameters with respect to the biodegradation of Mg-based stents. We would like to summarize the latest achievements as well as comment on the selection and use, test methods and the approaches to develop and produce Mg-based stents that are intended to perform clinically with an appropriate host response.

## Physiological Parameters

### Fluid flow and diffusion

**Figure 1.**
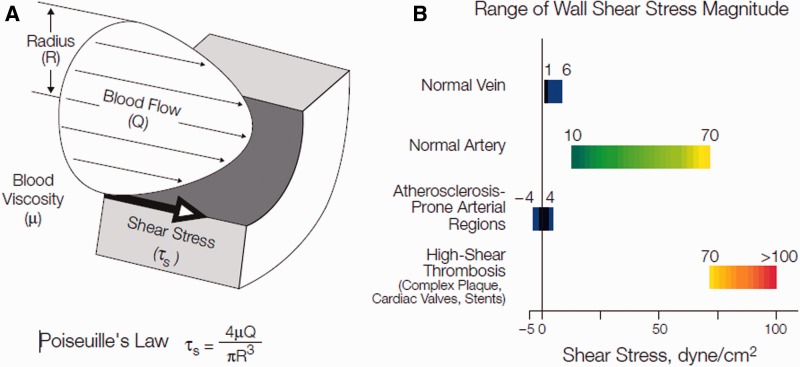
(**A**) Cross-sectional schematic diagram of a blood vessel illustrating hemodynamic shear stress, τ_s_, the frictional force per unit area acting on the inner vessel wall and on the luminal surface of the endothelium as a result of the flow of viscous blood. (**B**) Tabular diagram illustrating the range of shear stress magnitudes encountered in veins, arteries and in low-shear and high-shear pathologic states [23].

To effectively study, model and optimize absorbable metallic stents, characterizations need to be performed in hydrodynamic conditions that are found within vessel walls. The blood fluid drag force acting on the vessel wall is mechanotransduced into biochemical and biophysical signals that result in changes in vascular behavior and microenvironment around the stent [21]. The stent is exposed to the blood fluid at the initial stage of implantation as a result of fluid convection that dominates the interaction between blood and stent. In the succeeding stage, the intima grows over the stent surface when fluid diffusion dominates in the interaction between tissue and stent. The magnitude of flow-induced shear stress on the vessel walls can be estimated in most vasculature situations by Poiseuille’s law [22] (Fig. 1A). Various arteries have different values of peak and mean wall shear stress in the human body as shown in Table 1. The shear stress ranges from 1 to 6 dyne/cm^2^ in the venous system and between 10 and 70 dyne/cm^2^ in the arterial vascular network (Fig. 1B). The presence of a deployed stent in an artery may increase the shear stress to between 70 and 100+ dyne/cm^2^ [23]. Thus, it is important to analyze the effects of flow conditions in a biologically relevant range of flow conditions for the successful application of absorbable metals for vascular stents [24] and other clinical applications for stents such as trachea and ureter stenosis [25, 26].

**Table 1.** Peak and mean wall shear stress in typical vascular sites in healthy human subjects

Wall shear stress (dyne/cm^2^)	Carotid artery [27]	Coronary artery [28]	Suprarenal aorta [29]		Infrarenal aorta [29]		Femoral artery [30]
			Anterior	Posterior	Anterior	Posterior	
Peak	30.5 ± 5.2	12.4	48.0 ± 6.0	54.0 ± 11.0	33.0 ± 2.6	30.0 ± 7.0	41.4 ± 10.4
Mean	12.3 ± 2.1	6.8 ± 0.3	8.6 ± 3.2	10.4 ± 2.9	6.1 ± 2.6	4.7 ± 2.6	3.6 ± 1.6

Data are expressed as mean ± standard deviation. 1 dyne/cm^2 ^= 0.1 Pa = 0.1 N/m^2^.

**Figure 2.**
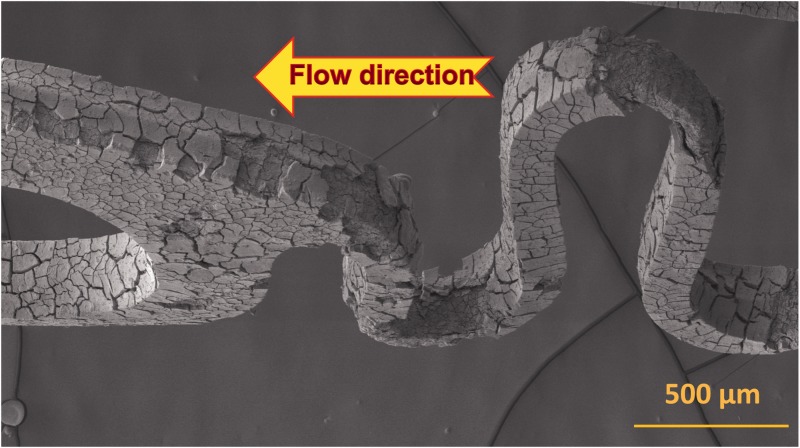
Scanning electron microscopy image of AZ31 stent at a shear stress of 0.56 dyne/cm^2^ in DMEM for 7 days. The arrow indicates the flow direction of corrosion medium [32].

The fluid flow has a significant impact on the degradation of absorbable metallic stents, including corrosion type, corrosion rate, corrosion products and local pH change. Shear stress accelerates the overall corrosion rate (including localized, uniform, pitting and erosion corrosions) due to the increase of mass transfer and mechanical force [31, 32]. Shear stress also increases the thickness of the uniform corrosion layer, the localized corrosion coverage ratios and depth, and the removal rate of corrosion products inside the corrosion pits [32]. For instance, the volume loss ratio (31%) of AZ31 stents at a shear stress of 0.56 dyne/cm^2^ was nearly twice that (17%) at static condition in Dulbecco’s modified Eagle’s medium (DMEM) after 7 days degradation [32]. Furthermore, flow direction made the corrosion product layer facing the flow direction peeled off from the AZ31 stent struts (Fig. 2) [32]. Additionally, flow can assist in maintaining the pH level around a basic physiological value, resulting in continuation of normal degradation behavior [33].

**Figure 3.**
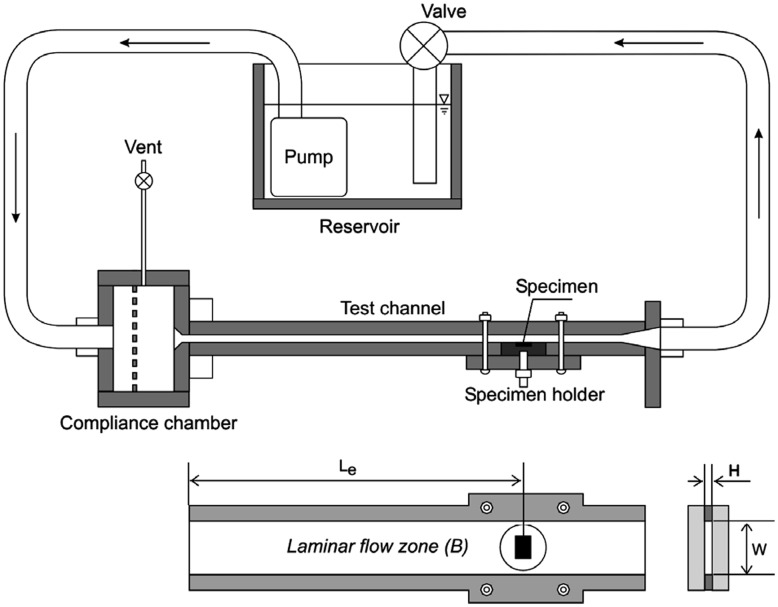
Schematic view of the dynamic test bench and close-up upside view of the test channel with specimen in place. Note: Le = length of laminar flow before reach specimen, W = width and H = height of the test channel [31].

To design the fluid dynamic experiment, the geometry of the test bench and the calculation of shear stress need to be considered delicately. In the case of the stent and tube samples, the wall shear stress calculated by Poiseuille’s law always represents the shear stress on the sample. Essentially, the highest shear stress on the stent occurs on the surface of struts facing the flow direction simulated using a three-dimensional (3-D) computational fluid dynamics (CFD) model. This highest shear stress value is more than twice the wall shear stress value [34]. In terms of the geometry, Poiseuille’s law is no longer applicable to the shear stress calculation of conventional plates in the lumen. Lévesque *et al*. [31] provided a good test bench for this case. The surface of the plate and the wall of the test channel have a smooth transition, resulting in a uniform shear stress zone on the sample surface. It avoids complex flow patterns by the interruption of the geometry of the plate (Fig. 3). Otherwise, there will be a need for a CFD model to calculate shear stress on the sample surface [32]. Additionally, the viscosity of DMEM with supplements for the *in vitro* test is 0.78 mPa s at 37°C [35], which is lower than that of blood (3.5 mPa s) [28].

**Figure 4.**
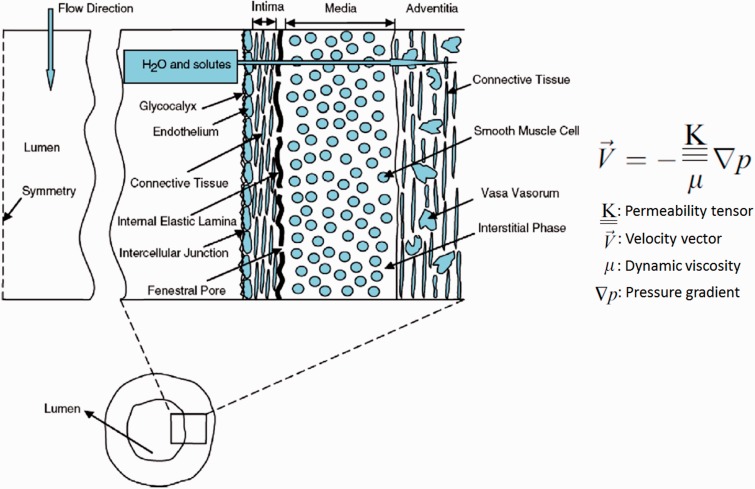
A schematic of the anatomical structure of an arterial wall [37].

After the intima layer covers on the stent [36], the stent is located between the intima and media. The degradation of the stent is dominated by water molecules and hydrophilic solutes and ions diffusion between vascular tissue and stent. These diffusion rates are related to the structure of the arterial wall as schematically shown in Fig. 4 [37]. The arterial wall is treated as a permeable channel which commonly consists of six layers: glycocalyx, endothelium, intima, internal elastic lamina, media and adventitia [37, 38]. Water molecules and hydrophilic solutes and ions permeate through interendothelial junctions by way of transportation utilizing fenestral pores that are embedded throughout the intima, internal elastic lamina and media sections of the arterial wall. Consequently, it is important to describe the nature of transportation of these molecules and ions through the arterial wall by way of permeation by utilizing porous media transport models. Fluid flow through porous media has been modeled by the conventional Darcy law which is a generalized linear relationship between the flow velocity ($\overset{\to }{V}$) and the pressure gradient (∇p) across a porous medium (Fig. 4) [39]. Furthermore, mass transfer across the arterial wall occurs via two mechanisms: convection associated with pressure-driven transmural flow and mass diffusion caused by concentration gradients. The molecular diffusion within the arterial walls is driven by solute concentration gradients. Building an *in vitro* simulation model of this case has some challenges, due to the compilation of the structure of the arterial wall and the difficulty of the artery culture *in vitro*. Therefore, the data from *in vivo* tests are more substantial and reliable.

### Composition of media

The overall bioactivity of Mg alloys is affected by the composition of the physiological solution. As a consequence of the complicated and adjustable *in vivo* environment, *in vitro* experiments require the analysis of various physiological corrosion media to elucidate the underlying interaction mechanism between Mg and microenvironment. It is based upon the primary interaction of inorganic ions and proteins with the Mg as they have a strong impact on cell/surface interaction. Jang *et al*. [40] investigated the effect of different inorganic ions on degradation (Table 2). It was reported that: (i) calcium is not present in the corrosion product layer when only Cl^−^ and OH^−^ anions are available; (ii) the presence of phosphate induces the formation of a densely packed amorphous magnesium phosphate corrosion product layer when ${\text{HPO}}_{\text{4}}^{{{}^{\text{2}}}^{-}}$ and Cl^−^ are present in solution; (iii) octacalcium phosphate and hydroxyapatite are deposited on the surface of the Mg alloy when ${\text{HPO}}_{\text{4}}^{{{}^{\text{2}}}^{-}}$ and Ca^2+^ are present together in NaCl solution (this coating limits localized corrosion and increases general corrosion resistance); (iv) addition of ${\text{HCO}}_{\text{3}}^{-}$ accelerates the overall degradation rate, which increases with increasing bicarbonate concentration; (v) the degradation rate in bicarbonate solution decreases in the presence of ${\text{HPO}}_{\text{4}}^{{{}^{\text{2}}}^{-}}$ ions due to the formation of insoluble hydroxyapatite on the surface when ${\text{HCO}}_{\text{3}}^{-}$ is present with Ca^2+^ and ${\text{HPO}}_{\text{4}}^{{{}^{\text{2}}}^{-}}$. Therefore, the inclusion of proteins in medium solutions is vital in terms of better mimicking an organic microenviroment *in vitro*, which will facilitate a more accurate understanding of the degradation processes. It has been reported that degradation slows down considerably when proteins are a part of the corrosion medium [41, 42]. This effect is explained as protein adsorption on the surface forming a corrosion-resistant layer. This suggests that the bioactivity of these materials *in vivo* will be strongly affected by the presence of biomolecules. Cell growth media such as DMEM or Eagle’s minimum essential medium are closer to physiological conditions found *in vivo* because of the addition of inorganic salts, amino acids and vitamins within the solution.

**Table 2.** Analytical methods used in studying the solid corrosion products of Mg and its alloys *in vivo* and *in vitro*

Methods	Functions	Reference
Optical microscopy	Morphology	[63]
Electron beam:	Morphology, distribution and composition	[63–65]
Scanning electron microscopy Energy dispersive x-ray Backscattered electron Density-dependent color		
Microscopic fourier transform infrared spectroscopy	Composition	[63, 64, 66]
*In situ* attentuated total reflectance Fourier transform infrared spectroscopy		
X-ray diffraction	Composition	[40]
Microtomography	Morphology and distribution	[67, 68]

### Gas diffusion

#### Carbon dioxide

CO_2_ plays a crucial role in the degradation of Mg [17]. The presence of CO_2_ might trigger several reactions that influence the degradation process. In terms of possible stent applications, the CO_2_ values in arterial and venous blood are 4.12% and 5.32%, respectively [43]. Furthermore, CO_2_ produces H_2_CO_3_ in aqueous environments, which dissociates into H_3_O^+^ and ${\text{HCO}}_{\text{3}}^{-}$. The latter, hydrogen carbonate, plays a critical role in the blood buffering system and maintaining a neutral pH of the corrosion solution. In parallel, ${\text{HCO}}_{\text{3}}^{-}$ produces ${\text{CO}}_{\text{3}}^{{{}^{\text{2}}}^{-}}$ and H_3_O^+^ in aqueous environments that can result in a basic pH and consequently have an impact on the degradation rate of Mg.

#### Oxygen

O_2_ plays a less prominent role in the degradation of Mg [17]. This lower level of influence of O_2_ on the degradation behavior might be associated with the fact that O_2_ does not change the pH and that it most likely does not directly interact with Mg. It has been recommended that the concentration of O_2_ within *in vitro* test conditions be reduced in an effort to more accurately replicate *in vivo* conditions because the content of O_2_ in many tissues is ∼1–7%, which is less than that of ambient air (21%) [44–46]. As a consequence, O_2_ is not important when 5% CO_2_ is present within the environment.

### pH

As Mg generates H_2_ and OH^−^ along the progress of its degradation reaction with medium, the pH of the fluid around Mg surface will increase. Although the instability of Mg occurs at pH values <11, the formation of soluble compounds with most inorganic ions inhibits the formation of magnesium hydroxide passive films in the biological environment [47]. These reactions result in an increase in both magnesium concentration and alkalinity near the stent surface, which may affect surrounding cellular and tissue function [48]. At local pH levels over 11, the formation of the magnesium hydroxide film on the Mg surface can create a barrier with the corrosion solution that slows the degradation process [24, 49]. In biological systems, the pH is regulated by the circulation of blood which keeps environment at normal pH levels. Therefore, in the design of *in vitro* degradation experiments, the degradation solution must be monitored and replaced with fresh medium on a consistent basis to ensure normal pH levels, that mimic the *in viv*o environment, are maintained. Additionally, the calcium phosphate complex as a passivation layer can be adsorbed on the surface under a high pH microenvironment [33]. Nevertheless, flow can keep pH at around basic physiological value, due to rapid ion diffusion. Therefore, the results from the dynamic fluid tests may be closer to that in vascular environments.

### Temperature

The effect of temperature in the human physiological range ∼35.8–37.2°C seems to be less important for Mg degradation [24]. It may affect the adsorption of proteins and thus the biological response.

In brief summary, these physiological parameters are separated as the first and second order relevance [17]. ‘First order relevance’ consists of parameters such as CO_2_, the presence of NaCl and NaHCO_3_ and temperature that directly influence the degradation rate. ‘Second order relevance’ consists of parameters such as O_2_ and proteins that influence the corrosion layer and the final corrosion products. These parameters might also influence the cell reaction *in vivo*.

## Current Methodologies and Characterizations of Biodegradation Behavior

### Corrosion rate

Mass loss (ML) *in vitro* experiments provide a benchmark for determining the actual amount of cumulative corrosion that has occurred for Mg alloy degradation. ML experiments typically have a simple setup in which the experiment requires only a sample, corrosion medium and an accurate microbalance. The ML procedure consists of the sample being placed in the corrosion medium for a specified period of time, after which the sample is removed and the resultant mass change is measured. Prior to measuring the final mass, a cleaning solution such as dilute chromic acid (180–200 g/l CrO_3_) for 5–10 min [50] is used to remove corrosion products from the surface. The corrosion rate based on this type of experiment is calculated in equation (1) [16]:

(1) $$CR=\frac{W}{At\rho }$$

where *CR* refers to the corrosion rate (mm/y), *W* is the weight loss of the sample, *A* is the exposure area, *t* is the immersion time, and ρ is the standard density. Although these type of tests are simple to set up and easy to operate, there are inherent limitations such as accounting of non-uniform corrosion as well as the inability to determine the mechanism behavior of Mg alloys.

Morphology analysis provides a visualized measurement for determining the corrosion rate based on the corrosive morphology and structure of the sample. The outward appearances and cross-sectional images of the corrosive samples can be reconstructed by X-ray computed tomography (CT). Corrosion rate depending on the reduction of the metallic volume after the corrosion can be calculated from the 3-D CT data. Assuming a uniform corrosion, the reduction of the implant volume could be converted into a corrosion rate by using a modification of equation (2) [16]:

(2) $$CR=\frac{\Delta V}{At}$$

where *CR* refers to the corrosion rate, Δ*V* is the reduction in volume that is equal to the residual alloy volume (without corrosion product volume) subtracted from the initial implant volume, *A* is the sample surface area exposed to corrosion and *t* is the exposure time. It must be noted that this method is a rough calculation without the consideration of nonuniform corrosion [32]. To obtain a more accurate analysis, the uniform and localized corrosion rates, which are the two major types of Mg corrosion, can be calculated by electron dispersive x-ray spectroscopy (EDX) and CT. The average uniform corrosion rates were calculated according to the thickness of the corrosion product layer divided by exposure time. The thickness of the corrosion product layer can be measured by EDX line analysis. The local corrosion coverage ratio of the sample at different depths can be calculated from 2-D images using Image-Pro® Plus.

Hydrogen evolution experiments are based on the amount of H_2_ gas that evolves from the interaction between Mg sample and the environment and this type of testing is used to artificially determine mass loss at any point in time. A typical setup is similar to that of a standard mass loss test. The sample is immersed in the corrosion medium and a collector is placed in the medium directly above the sample to collect H_2_ gas that is produced. A collector consists of an inverted funnel and burette filled with corrosion medium [51]. Many Mg corrosion studies employ the collection of gaseous H_2_ on the basis that the primary cathodic reaction (2H_2_O + 2e^−^ → 2OH^−^ +H_2_ [52]) is an index to the rate of anodic dissolution (where at open circuit, *I*_anodic_ = *I*_cathodic_) [15]. Thus, the evolution of 1 mol of hydrogen gas (22.4 l) directly corresponds to the dissolution of 1 mol of Mg (24.31 g). The principle behind interpreting H_2_ evolution is that the volume of H_2_ gas is deemed to be equivalent to the mass loss of the Mg [19]. However, the majority of the literature concerning hydrogen evolution has not reported the theoretical 1:1 ratio of hydrogen evolved to actual mass loss [19]. This may be attributed to some inadequacies of the hydrogen collection method, given that (i) hydrogen collection may be inefficient in cases where experiment design is not ideal, (ii) the solubility of hydrogen in water varies significantly relative to sea level and temperature, and (iii) studies rarely pre-saturate the corrosion medium with hydrogen [15]. Therefore, it is important to couple mass loss measurements with hydrogen evolution experiments as a way of vetting the results.

Electrochemical technique is crucial for determining and quantifying the corrosion mechanism of Mg alloys. A commonly used technique is the utilization of direct current polarization (DCP) test, which is preceded by a set period of exposure time where the open circuit potential (OCP) is recorded. This allows the material to ‘stabilize’ with the electrolyte and reach a near steady potential. After OCP stabilization, voltage is swept at a controlled rate (e.g. 1 mV s^−1^) between different pre-set potentials by regulating the current flowing between the working (Mg) and counter (inert metal) electrode. The initial voltage is nominally set to commence at values more negative than (i.e. cathodic to) the OCP, and the scan proceeds to increasingly positive values (that are anodic to the original OCP) [19]. The DCP results show as a Tafel curve, which provides thermodynamic information on corrosion potential (*E_corr_*), kinetic information from the corrosion current density (*i_corr_*) as well as relative anodic and cathodic reactions. In this regard, it is the only method which can reveal the relative anodic and cathodic contributions that lead to an instantaneous corrosion rate. The corrosion rate, *CR*, in millimeter per year can be determined from equation (3) [53]:

(3) $$CR=\text{3}.\text{27}\times 10^{-3}\frac{i_{corr}\;EW}{\rho }$$

where *EW* is the equivalent weight of the corroding species in grams and ρ is the density of the corroding material in g/cm^3^. Assuming uniform corrosion, *i_corr_* is converted to a corrosion rate (in terms of penetration) and therefore the DCP results do not typically yield an absolute corrosion rate for Mg, but rather are indicative of the severity of the corrosion that is occurring at a select point in time, in terms of current density. However, uniform corrosion is seldom in the degradation of Mg alloys and thus none of the methods described in current reports provides an absolute prediction of the corrosion rate. Conversely, the corrosion rate expressed in current density is highly accurate and can be considered to have the highest resolution of all methods described herein, although the current could be originating from a number of local sites on the surface. The method, however, is short-term and destructive in nature. As such, wherease potentiodynamic polarization is indispensable in understanding the mechanistic origins of corrosion rate, it may not serve as a good index to long-term corrosion rates.

Electrochemical impedance spectroscopy (EIS) is another technique that is used to characterize the surfaces of Mg sample using the frequency response of AC polarization [54, 55]. EIS uses a range of low magnitude polarizing voltages that cycle from a peak anodic to peak cathodic voltage using a spectra of voltage frequencies. Capacitance and resistance values are obtained for each frequency and can then be used to illuminate a number of phenomena and properties of the Mg surface. An appreciated understanding can be summarized in that faithful determination of corrosion rate was possible when the EIS determined polarization resistance (*R_p_*) parameter at the zero frequency limit was used [15]. The *R_p_* is inversely proportional to the *i_corr_* as described by the Stern–Geary relationship [53]:

(4) $$i_{corr}=\frac{\beta_{a}\beta_{c}}{2.303R_{p}(\beta_{a}+\beta_{c})}$$

where $\beta_{a}$ and $\beta_{c}$ are the anodic and cathodic Tafel slopes (as described and qualified further below) [56–58]. EIS determined corrosion rate can be transferred by the *i_corr_* based on equation (3). The primary benefits of conducting EIS on Mg alloys in an electrolytic solution are the identification and quantification of the formation behavior of corrosion layers produced by the corrosion process. However, EIS results have possible limitations in the fact that they can be affected by the continuation of Mg dissolution at low frequencies and the chosen equivalent circuit. Consequently, to be correctly employed, EIS requires a deep understanding of the corrosion processes that are taking place and how they might be best modeled.

Combined experiments are developed to take advantage of the relative merits of each testing method. King *et al*. [15] designed to permit *in-situ* EIS and H_2_ collection, along with subsequent post-mortem mass loss. The results provided an excellent correlation to mass loss, hydrogen gas collection and EIS-estimated polarization resistance. Doepke *et al*. [59] developed a real-time monitoring of the solution soluble corrosion products Mg^2+^, OH^−^ and H_2_ during immersion tests. This instrumentation was also developed to record electrochemical impedance spectra simultaneously in the same solution to monitor changes in the Mg samples.

### Corrosion products

**Figure 5.**
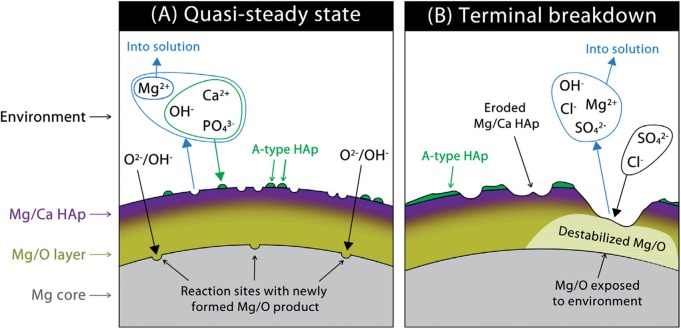
Schematic showing transformations supposed to be taking place in the quasi-steady state (**A**), including apatitic ion exchange and reactions at the Mg–Mg/O interface, and during the terminal breakdown stage (**B**), dominated by dissolution of the Mg/O layer upon exposure to the arterial environment [63].

Characterization of the explanted stent materials and identification of the products thereby generated are critical to the maturation of this class of absorbable implants. Various methods have been used to analyze the corrosion products of Mg and its alloys *in vivo* and *in vitro* (Table 2)*.* The morphology, distribution and composition of corrosion products can be comprehensively analyzed. Corrosion products of Mg corrosion have become common knowledge, such as the general tendency for Mg to form an oxygen-bearing product: MgO, Mg(OH)_2_, a Mg carbonate or a mixture thereof [60, 61]. Bowen et al. indicated the gradual replacement of Mg by calcium and phosphorus in vivo as schematically shown in Fig. 5 [63]. Several in vivo works mentioned that calcium and phosphorus formed on the Mg surface as well [8, 11, 60, 62].

Hydrogen (H_2_), as a gaseous corrosion product, has always been used as an evaluation parameter of Mg corrosion. Many researchers assume that the formation of gas cavities after implantation of Mg alloys contain primarily hydrogen because it is in aqueous media. However, Kuhlmann *et al*. [69] reported a direct monitoring method of hydrogen concentration was developed using an amperometric hydrogen sensor and mass spectrometric measurements for Mg alloy disks in mice over a period of 10 days. The results showed that the gas cavities contained only a low concentration of hydrogen gas, even shortly after formation of the cavities, which means that hydrogen must be exchanged very quickly after implantation. Therefore, the fate of H_2_ *in vivo* is worth of attention.

The local chemical reactions on the interface are of great importance for understanding the intricate mechanisms of Mg corrosion. Lamaka *et al*. [70] developed ion-selective microelectrodes for mapping local activity of H^+^ and Mg^2+^ by scanning ion-selective electrode technique as well as local ionic current density measurements by scanning vibrating electrode technique over the surface of Mg alloy in aqueous chloride containing solution. The local concentration of Mg^2+^ ions dissolved due to corrosion from artificial defects and pH changes together with anodic and cathodic currents can be measured by combination of two selective and one vibrating microelectrodes.

### Corrosion types

There are varvious corrosion types during Mg degradation process [71], including uniform corrosion [72, 73], localized corrosion [72, 73], flow-induced corrosion [31, 74], erosion corrosion [75], galvanic corrosion [76, 77], pitting corrosion [78, 79], stress corrosion [80, 81], atmospheric corrosion [82, 83], hydrogen cracking [84, 85] and intergranular corrosion [86, 87]. It is worth to note that localized corrosion is always a source of stent fracture.

To estimate the corrosion types, microscopic view of investigated surface areas is a direct method to detect changes to surface morphology. Some complementary electrochemical techniques also can be used. The prediction of corrosion activity, corrosion rate and the type of corrosion process (uniform, pitting or filiform corrosion) can be achieved, based on the corrosion current density and polarization resistance from the potentiodynamic curves. The shape of the curve gives an indication about the form of corrosion taking place at the surface, for example, a constant current range indicates the formation of a passive layer; a symmetric logarithmic curve around the corrosion potential indicates clearly the situation of uniform corrosion; pitting or crevice corrosion can be assessed based on the shape of the anodic part of the *i* versus *E* curve after the passive layer has been disrupted [88]. In EIS spectra, the inductive loop at low frequencies of Nyquist plot indicates pitting corrosion of the Mg alloy as well.

### Mechanical behaviors under corrosion

**Figure 6.**
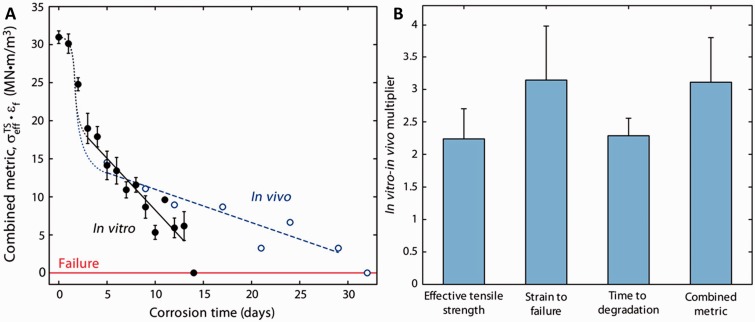
(**A**) Trend observed for the combined mechanical metric for *in vivo* (empty circle) and *in vitro* (filled circle) samples, with the two relevant linear trends (*in vivo* and *in vitro* dotted and solid, respectively). (**B**) Visual presentation of the various mechanical *in vivo–in vitro* multipliers. Error bars are indicative of standard error [89].

When designing absorbable Mg implant devices, the implant material has to achieve certain corrosion behaviors to assure proper mechanical behaviors during the entire healing process. More specifically, the implants must be able to mechanically support the damaged tissue site for at least 3–6 months and then completely degrade and removed from the healed tissue site in 7–24 months. Therefore, the mechanical properties *in vivo* are vital to the success of an absorbable stent. An *in vitro–in vivo* correlation of mechanical analysis is proposed in Fig. 6 by Bowen *et al*. [89] for the physiological corrosion of pure Mg wires in the arterial walls of rats (*in vivo*) and in static cell culture media (*in vitro*). In tensile testing, the *in vivo* degradation was 2.2 ± 0.5, 3.1 ± 0.8, 2.3 ± 0.3 and 3.1 ± 0.7 times slower than corrosion *in vitro* in terms of effective tensile strength, strain to failure, sample lifetime and combined metric (defined as strength multiplied by elongation), respectively.

A well-established method to analyze the potential mechanical integrity of a stent is through computational modeling. Computational modeling of the degradation process and the associated mechanical integrity degeneration are investigated in a finite element (FE) framework. A continuum damage model was developed for Mg alloy stents considering superposition of stress corrosion and uniform corrosion [90, 91]. Stress corrosion will take place at the predicted locations with high residual stress, where the stent ring will break earlier than at other locations; uniform corrosion will cover the whole exposed sample surface and attack the stent in a ‘layer-by-layer’ manner [92].

As the model depicted, the beginning of Mg alloy degradation was concentrated mainly at more deformed locations after stent expansion. The degradation proceeded with the mass loss of the outer surface of the stent, associated with the decreasing mechanical integrity of the stent. The optimized Mg alloy stent, with thicker strut than the Magic stent design [90], showed decreased maximum principal stress after recoil (163 MPa) and increased half normalized time of vessel recoil [90]. Grogan *et al*. [93] developed a phenomenological corrosion model in a FE framework to predict the loss of stent mechanical integrity using both pitting corrosion and uniform corrosion models. Pitting corrosion attacks led to a non-uniform breaking down of the stent geometry while the uniform model predicted the homogeneity. The experimental results were in harmony with the numerical results using the pitting corrosion model. Grogan *et al*. [94] further developed an Abaqus the arbitrary Lagrangian–Eulerian adaptive meshing method to model the diffusion-controlled corrosion of a 3-D absorbable metal stent geometry. The assumption of diffusion is based on observations of the formation of stable layers of corrosion products in the body [60, 63, 95] or tissue layers [96] and the known diffusion-controlled corrosion process associated with stable corrosion product layers [97–99] for the long-term device corrosion. Assuming that the corrosion rate is governed by the diffusion of Mg^2+^ in solution, it is predicted that the mass loss rate from the stent is inversely proportional to the square root of immersion time. It is predicted that the mass loss rate is proportional to the saturation concentration of Mg^2+^ in solution and is related to the diffusivity of Mg^2+^ in solution through a power law behavior, where doubling the diffusivity increases the mass loss rate by a factor of ∼1.48. The physical model developed here is computationally efficient and can serve as a useful accompaniment to existing phenomenological models used in the analysis and design of absorbable metal stents.

## Summary

Various physiological factors can alter the biodegradation performance and subsequent testing methods in determining the feasibility of Mg-based stents for vascular applications. In this article, we strive to identify and test the relevant microenvironment and parameters in test systems as well as provide a summarization of accurate test methods with the comments on the selection and use. Examples include, understanding the degradation behavior of Mg based on the possible active interactions with a biological system, which will completely alter the environment around a stent, will eventually lead to the knowledge-based design of application-specific stent materials. Mimicking the environment of the vessel repair process is crucial in creating an effective test design and consequently there are a variety of factors that must be considered. The degradation tests should be performed in hydrodynamic conditions that include the consideration of flow conditions in the initial stage and the diffusion condition in the succeeding stage. Composition of the corrosive media, CO_2_ levels and pH value have a strong influence on the degradation of Mg, whereas O_2_ and temperature play a less prominent role.

This review highlighted the assortment of degradation assessments in an effort to expose the general development of physiologically relevant *in vitro* techniques that are needed to give better insight and possible prediction of the *in vivo* biodegradation behavior of Mg-based stents. Hydrogen collection, mass loss, direct-current polarization, electrochemical impedance spectroscopy and morphology analysis can all be used to not only get a holistic view of the degradation of Mg-based stents, but can also be used to support the accuracy of the corrosion data produced from each individual technique. Additionally, combining these experiments can also simultaneously analyze corrosion types, corrosion morphologies, corrosion products and hydrogen evolution. When designing absorbable Mg implant devices, the implant material has to achieve certain corrosion behaviors to assure proper mechanical behaviors during the entire healing process. Computational model have been developed to serve as a useful accompaniment to existing mechanical testing of absorbable metal stents. More research is being done in the exploration of improving the standardization process for *in vitro* testing methods, and it is necessary to establish *in vitro–in vivo* correlation of biodegradation behavior of Mg-based stents.
